# Model based estimation of QT intervals in non-invasive fetal ECG signals

**DOI:** 10.1371/journal.pone.0232769

**Published:** 2020-05-11

**Authors:** Namareq Widatalla, Yoshiyuki Kasahara, Yoshitaka Kimura, Ahsan Khandoker

**Affiliations:** 1 Next Generation Biological Information Technology, Tohoku University Graduate School of Biomedical Engineering, Sendai, Japan; 2 Advanced Interdisciplinary Biomedical Engineering, Tohoku University Graduate School of Medicine, Sendai, Japan; 3 Healthcare Engineering Innovation Center (HEIC), Department of Biomedical Engineering, Khalifa University, Abu Dhabi, UAE; University of Tampere, FINLAND

## Abstract

The end timing of T waves in fetal electrocardiogram (fECG) is important for the evaluation of ST and QT intervals which are vital markers to assess cardiac repolarization patterns. Monitoring malignant fetal arrhythmias in utero is fundamental to care in congenital heart anomalies preventing perinatal death. Currently, reliable detection of end of T waves is possible only by using fetal scalp ECG (fsECG) and fetal magnetocardiography (fMCG). fMCG is expensive and less accessible and fsECG is an invasive technique available only during intrapartum period. Another safer and affordable alternative is the non-invasive fECG (nfECG) which can provide similar assessment provided by fsECG and fMECG but with less accuracy (not beat by beat). Detection of T waves using nfECG is challenging because of their low amplitudes and high noise. In this study, a novel model-based method that estimates the end of T waves in nfECG signals is proposed. The repolarization phase has been modeled as the discharging phase of a capacitor. To test the model, fECG signals were collected from 58 pregnant women (age: (34 ± 6) years old) bearing normal and abnormal fetuses with gestational age (GA) 20-41 weeks. QT and QTc intervals have been calculated to test the level of agreement between the model-based and reference values (fsECG and Doppler Ultrasound (DUS) signals) in normal subjects. The results of the test showed high agreement between model-based and reference values (difference < 5%), which implies that the proposed model could be an alternative method to detect the end of T waves in nfECG signals.

## Introduction

Many heart defects and complications start developing during the prenatal period [1, 2]. It is estimated that around 1 out of 125 babies develop congenital heart defects before their birth [1]. Some of the born babies may suffer from minor heart defects that go undiagnosed for years [1]. For example, intrauterine growth restriction (IUGR), which affects around 3%—10% of pregnant women, has been associated with several cardiovascular diseases that develop during adulthood [2]. Therefore, to reduce the number of cardiovascular complications, fetal heart rate (fHR) monitoring has grown to be a vital procedure for pregnant women [3].

Currently, there are several invasive and non-invasive techniques that are used for monitoring fetal cardiac function. Fetal magnetocardiography (fMCG), fetal electrocardiogram (fECG) and Doppler Ultrasound (DUS) are examples of currently used techniques to monitor fetal health [4, 5]. fMCG is a non-invasive technique and the magnetic equivalent to fECG [6, 7]. fMCG is known for its high accuracy [5], however, it is very expensive and requires special rooms [7]. Furthermore, fMCG requires the need for highly specialized equipment and its current practice is available in a few institutions worldwide [8].

DUS is a non-invasive method that detects cardiac activity of the fetus to calculate the HR [4]. DUS involves risks because, up until this date, it is not confirmed if exposure to ultrasound is completely safe for the fetus [9]. Also, sometimes, DUS fails to provide accurate assessment of fHR. For accurate assessment of fHR, fetal scalp ECG (fsECG) is usually used [4]. In contrast to DUS, fsECG can provide more information about the function of the heart. fsECG has been used in STAN monitor to estimate the ST segment and the amplitude ratio T/R [4, 9]. STAN analyzers have decreased the number of cesarean and hypoxic ischemic encephalopathy death cases at the St George’s Maternity Unit [9].

Despite the accuracy of fsECG, it is considered risky because it may cause infection [10]. Furthermore, it can be used for short-term monitoring and during labor only [10, 11]. Another safer approach involves using non-invasive fECG (nfECG) which can be used before and after labor [11]. nfECG can be collected, non-invasively, by attaching electrodes on the abdominal surface of the mother. The collected signals from the maternal abdominal surface are then processed to separate maternal ECG (mECG) from fECG [12]. Signals extracted by nfECG are usually accompanied with high noise which makes it hard to detect low-frequency waves such as T waves. T waves are generally hard to detect due to their low amplitudes compared to R peaks. In addition, they occasionally overlap with P waves [13].

Determining the end of T wave is vital for the assessment of ST and QT intervals. Evaluation of ST segment is important to diagnose hypoxia and ischemia [4, 11]; and evaluation of QT interval is useful in diagnosing sudden infant death syndrome and intrapartum hypoxia [11, 14]. Therefore, techniques or algorithms, which can pinpoint end of T waves in nfECG records, can facilitate non-invasive monitoring of fetal cardiac function. To our knowledge, there is no study that has been dedicated to estimating end of T waves in nfECG signals. This study proposes a model to pinpoint end of T waves in normal nfECG records. The model estimates T-end locations based on R peak locations only. Some abnormal fECG cases were included in this study to investigate how the model changes with the presence of abnormalities.

## Model description

Early models of the heart action potential (AP) have been stemmed from the Hodgkin-Huxley (HH) model [15]. The HH model provides an electric circuit representation for the electrical performance of a nerve axon [16]. In the HH model, membranes are represented as capacitors, and sodium and potassium ions are represented as currents [16]. *D. Noble* [15] discusses a cardiac model based on HH that investigates sodium current activity in the Purkinje fiber. The model in [15] shows that the sodium current exhibits a curve similar to the charging and discharging of a capacitor in an RC circuit [17].

The function of sodium channels is mostly dominant at the start of the AP. As AP progresses, the number of open sodium channels decreases, and the number of open potassium channels increases [18]. Potassium channels are the main contributors to the repolarization phase, and the decay they exhibit at the end of the phase is very similar to an exponent decay [18]. Due to the similarities between the graph of the cardiac AP or ventricular AP and the graph of charging and discharging of a capacitor in an RC circuit, a mathematical model based on the RC circuit, was developed to pinpoint T-end locations. Since a T wave indicates the repolarization phase in the AP [18], the developed mathematical model addresses only the repolarization phase. In this study, the repolarization phase has been modeled as the discharging phase of a capacitor. In an RC circuit, the discharging phase of a capacitor is given in, (1) [17].

$$V\left(t\right)=v_{0}\;e^{\frac{-t}{RC}}$$ (1)

where *t* is the time in seconds, *v_0_* is the initial voltage in volts, R is the resistance in ohms, C is the capacitance in farads. (1) was used as the base to develop the mathematical model to calculate T-end timings [17]. RC circuits can be used as passive low-pass or high-pass filters. When designing such filters, a cut-off frequency *f_c_* is usually calculated. The relationship between *f_c_* and RC is shown in (2) [17].

$$\text{RC}=\frac{1}{2\pi f_{c}}$$ (2)

In fECG, frequency is equivalent to HR, and HR is the inverse of RR interval [19]. Therefore, the time constant RC in (1), was replaced by a variable based on RR interval and the expression in (2). The model in this study calculates T-end points beat by beat, thus, RR interval was considered one beat at a time. Also, to facilitate calculations of T-end points, *v_0_* in (1), was set to 100. The final equation that was used to calculate the repolarization phase is given in (3). RR interval and *t* in (3) were taken in milliseconds (ms) to facilitate calculations. Also, in this model, calculations of the repolarization phases start from R peaks.

$$R\left(t\right)=100\;e^{\frac{-2\pi t}{RR}}$$ (3)

(3) shows that the repolarization phase depends on RR intervals. In fact, the relationship between the ventricular repolarization phase and the HR is supported in [20, 21]. *F. Vahedi et al*. [20] address that in the absence of conduction abnormalities, an increase in HRs causes a reduction in the heterogeneity of AP morphologies, ventricular depolarization instant, T-areas and T-amplitudes. *D. Bernardo et al*. [21] state that in normal subjects, higher HRs result in shortening in AP and repolarization phase. After calculating the repolarization curves for beats, the mean values of R(t) were used as the base to develop another equation to determine an interval in which an end of a T wave is expected to exist. The final equation was developed after investigating how the mean value of R(t) associates with heart rates in several fECG beats. Based on the mean value of R(t), a constant *k* is calculated to obtain an interval to calculate an end of a T wave. The constant *k* is calculated using (4) where *x* is the reciprocal of RR in seconds (s) for one beat.

$$k=|\frac{mean(R(t))}{x}-\frac{6\pi }{x^{2}}|$$ (4)

Using (4), an interval within R(t) is calculated to find a value for an end of a T wave. The value was obtained by taking the median of the interval described in (5).

EndofTwave=median(k-0.5<R(t)<k+1) (5)

**Fig 1:**
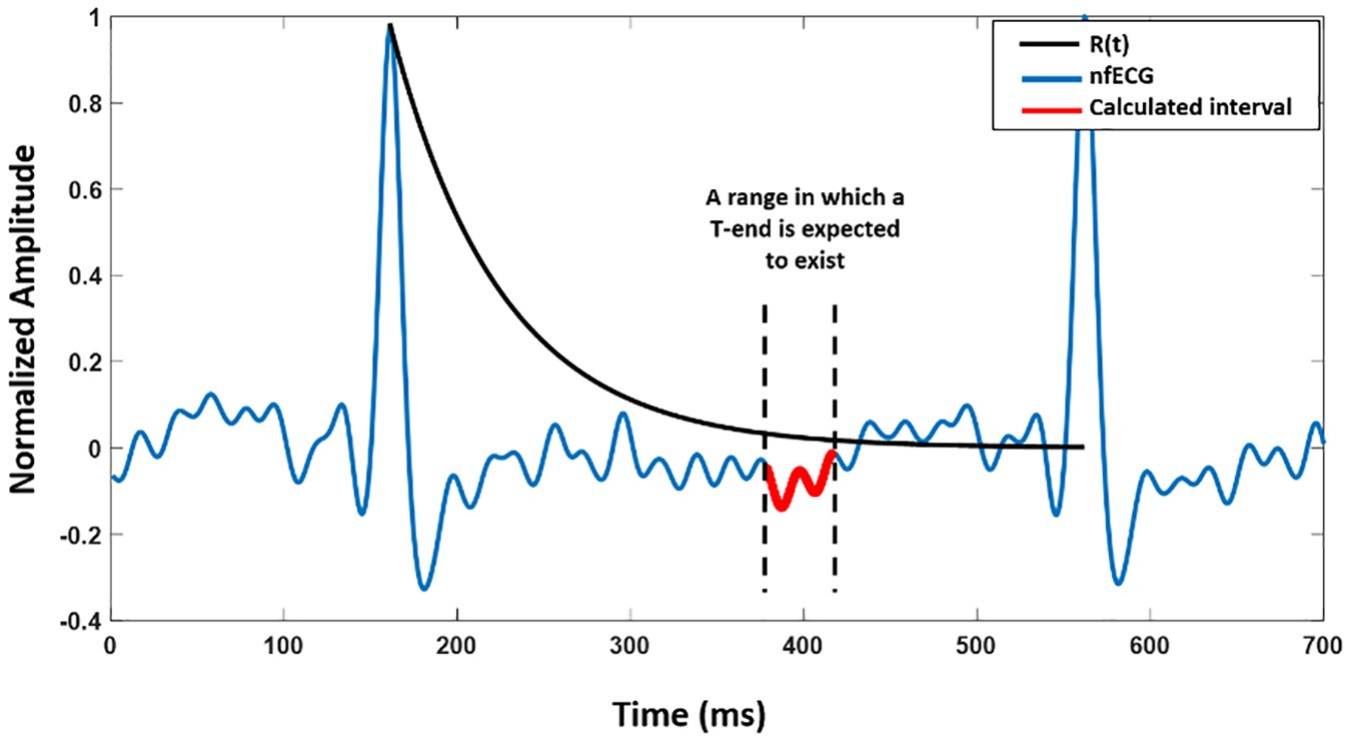
An example for T wave end estimation in one beat of nfECG. After plotting R(t), using (3), an interval in which a T wave end is expected to exist was calculated; by taking the median of the interval, one point for an end of a T wave was calculated.

An example of a beat from a nfECG with R(t), and estimated interval for T wave end is in Fig 1. By taking the median of the interval, an end of a T wave was determined.

## Materials and methods

### Data collection

58 pregnant women (age, (34 ± 6) years old, gestational age (GA): 20-41 weeks were recruited at Tohoku University Hospital after obtaining their written informed consent. The study protocol was approved by the Tohoku University Institutional Review Board. Out of the 58 pregnant women, 49 had healthy fetuses (GA: 20-41 weeks) and 9 had unhealthy or abnormal fetuses (GA: 24-36 weeks). The abnormalities were fetal tachycardia, fetal bradycardia and long QT syndrome (LQTS), heart anomaly, heart failure, IUGR, placental dysfunction and vasa previa. Among the 58 pregnant women, mECG, DUS and nfECG (from abdominal leads) were collected from 55 women; however, mECG, DUS, nfECG and fsECG (from fetal scalp) were collected from the rest of the 3 pregnant women at GA of 38-41 weeks. The records were collected simultaneously for 20 minutes The nfECG signals were collected at a sampling rate of 1 KHz by attaching 12-electrodes on the abdominal surface of the mother. DUS records were collected at 1.15 MHz from Ultrasound Transducer. fsECG records were collected by attaching an electrode to the fetal scalp.

### Signal processing

The raw signals collected from the maternal abdominal surface, have been processed in MATLAB to extract fECG. Blind source separation with reference (BSSR) was applied on the signals to extract nfECG records. The separation method, BSSR, is explained in detail in [22]. Extraction of nfECG signals of the whole 20 mintues period was not possible in all records, because they had high noise which affected the extraction of fECG signals. Some records had clear nfECG signals but noisy DUS records, therefore, they have not been included. In addition, beats with unclear T waves in fsECG were not included in this study. Of the 3 fsECG records, one record was not used for validation due to the noisy signal. Records of fsECG had base line and high frequency noise. The base line and high frequency noise were filtered in MATLAB using the discrete wavelet transform. fsECG signals have been decomposed into 10 levels using the Daubechies wavelets (db4). The baseline noise was filtered by removing levels 7-10 (1–7.8 Hz), and high frequency noise was removed by removing levels 1-4 (63–1000 Hz).

### fECG feature extractions

A MATLAB code has been developed to identify the locations of R peaks in fECG records. The R peaks were identified based on a threshold value that was changed based on R peak amplitudes. Based on R peak locations, RR intervals were calculated to estimate the curves of R(t) using (3). After calculating R(t), an interval in which a T wave is expected to exist was calculated through (4). The median of the interval was calculated to determine one point as an end of a T wave using (5). Q values have been identified manually, and mostly; they have been regarded as the lowest point that preceded an R peak for consistency. Similarly, values of aortic closing (Ac) in Doppler signals have been recorded manually. In order to identify the timing of Ac in M-mode Doppler images, durations with visible Ac have been identified. After that, the M-mode images were aligned with the simultaneously recorded nfECG signal. The value of Ac was determined by drawing a straight line from the Ac timing in the doppler image to the nfECG signal.

### Validation of the model-based results

Due to the lack of reliable nfECG databases with annotated T waves [9], other types of records have been used to validate the results obtained from this study. The other records that were used for validation were DUS and fsECG. In DUS records, end of T waves is equivalent to the Ac as mentioned in several literature [2, 23–27]. In this study, the time duration in which an Ac point was identified between two R peaks was approximately 170 ms–330 ms from an R peak. Previous studies identified the same interval as 180 ms–260 ms in [24] and as 140 ms–260 ms in [23]. The duration in this study was higher due to the presence of abnormal fECG cases. The end of T wave in fsECG was measured by drawing a tangent to the T wave and a baseline and the intersection of the tangent with the baseline was considered as the end timing of a T wave [28].

### Calculation of QTc

Bazett [29], Fridericia [30], Framingham [31], and Hodges [32] are commonly known QTc formula, nevertheless, all of them are controversial [33, 34]. Bazett is the most commonly used formula for correcting QT, however, its correction becomes unreliable when RR deviates from 60 bpm [35–37]. In our study, we attempted to correct fetal QT using the four formula, and we found that Framingham and Friderica provided consistent results. *J. Wernicke et al*. [38] performed QTc analysis on children and adolescent and they suggested a new formula, QTc = QT/*RR*^*0.38*^, which is close to Fridericia’s. Another study, by *D. Phan et al*. [39], discusses QTc analysis on infants and young children and it shows that Fridericia provided more consistent results over Framingham. Therefore, in this study, Fridericia was used for QTc calculation.

## Results

Analyses have been performed one minute at a time to identify the reference values for T-end points per beat. Reference T-end points were identified from fsECG records and DUS records. Therefore, only periods that had visible T waves in fsECG records or visible Ac in DUS records have been considered in this study. The total number of beats that have been filtered for analysis was 25,334 beats. Of the 25,334 beats, reference T-end points were identifiable in 19,110 beats.

**Fig 2:**
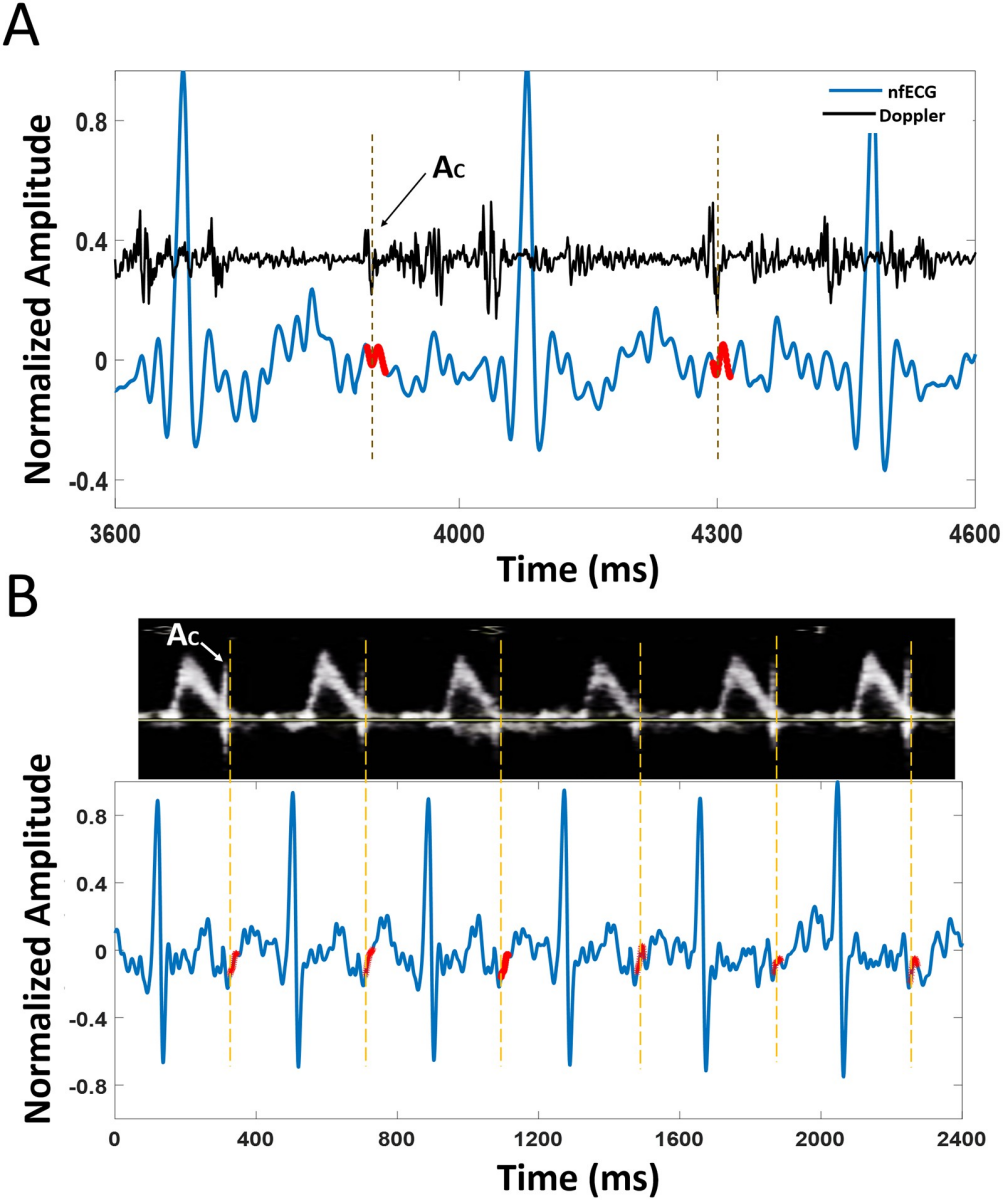
A: A normal nfECG signal (blue graph) is simultaneously plotted with a Doppler signal (black graph). The estimated T-end points (red dots) are compared with the Ac points in a simultaneously recorded Doppler signal. Ac timings are used to pinpoint T-end timings because it is hard to locate them in the noisy nfECG signal. B: A normal nfECG signal (blue graph) is simultaneously plotted with a M-mode Doppler image. The estimated T-end points (red dots) are compared with the Ac points in a simultaneously recorded Doppler image.

**Fig 3:**
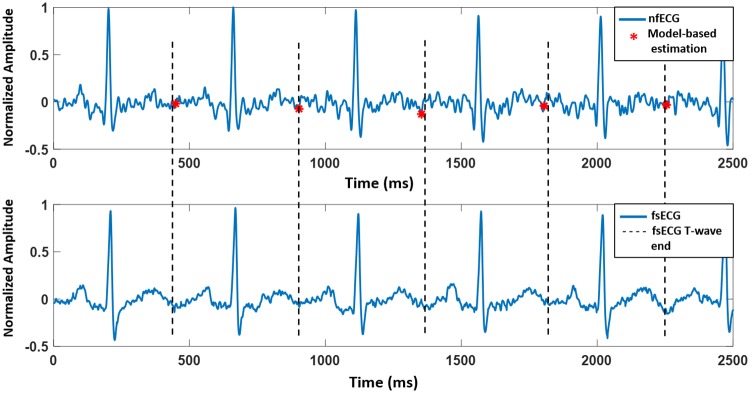
Model-based estimation of end of T waves validated by a fsECG signal. The above figure shows a tracing for nfECG with model-estimated end of T waves (red asterisk). The signal at the bottom shows a tracing of fsECG recorded simultaneously with nfECG. The dashed lines indicate the end of T waves of fsECG. End of T waves in fsECG were measured after drawing a tangent line at the T wave. The intersection of the tangent line with the baseline was considered the end timing of a T wave.

**Fig 4:**
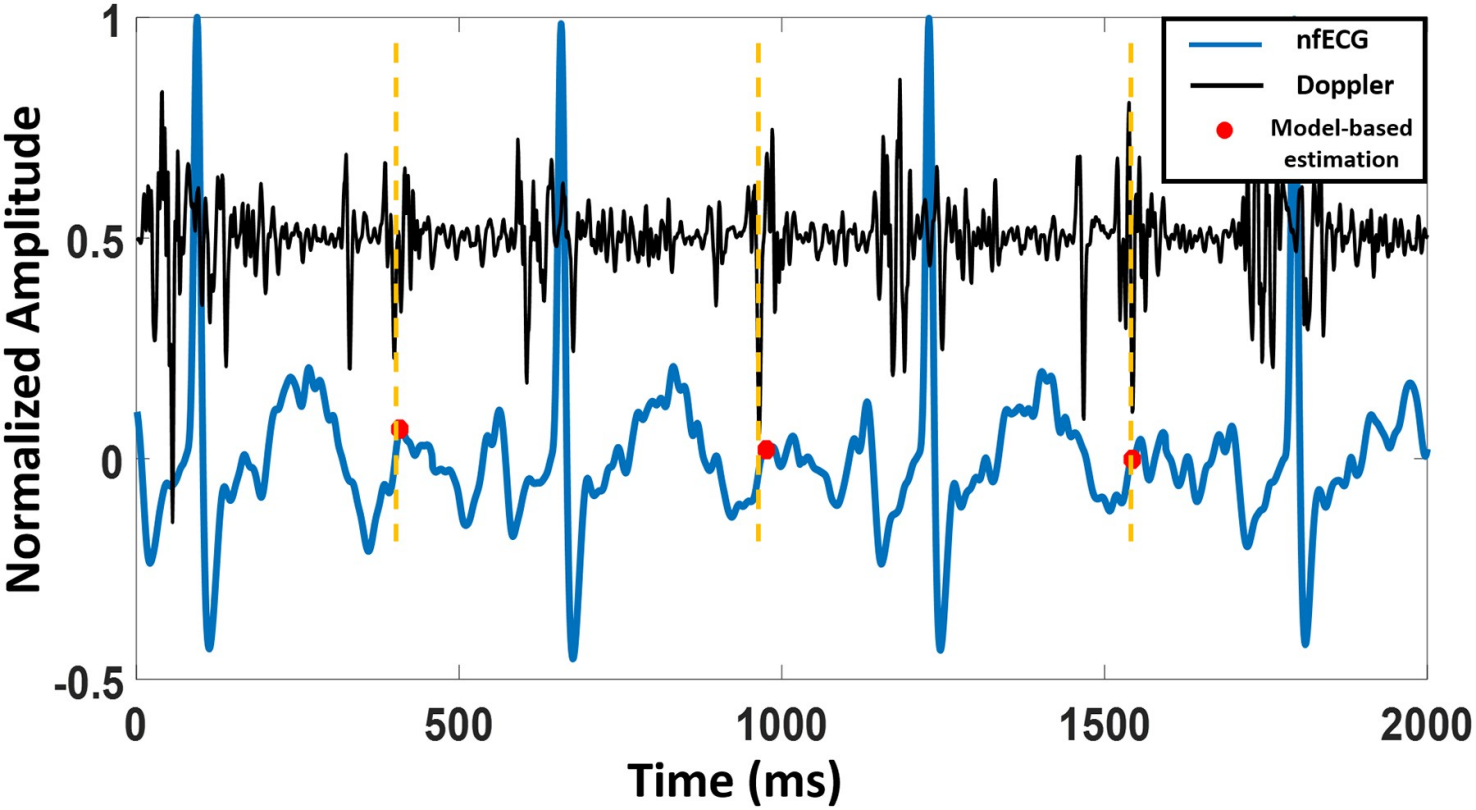
nfECG (blue graph) is plotted simultaneously with a Doppler signal (black graph) to locate T-end points. The figure shows signals for a subject that suffers from bradycardia and LQTS.

T-end points estimated by the model were compared with the reference values from a M-mode Doppler image, a Doppler signal or a fsECG signal. Doppler images were available for three subjects only, one normal and two abnormal (tachycardia and vasa previa). Fig 2 shows examples of model-based estimation of end of T waves for normal nfECG. The estimated values are compared with the Ac timings of a Doppler signal and a M-mode Doppler image. Fig 3 demonstrates an example of a model-based estimation validated by a fsECG record and Fig 4 shows an example for the LQTS case.

The mean, standard deviation (std) and root mean square error (RMSE) for QT and QTc of normal and abnormal subjects have been calculated to validate the results obtained from the model. A summary of the overall results is in Table 1 and a detailed result for the abnormal cases is in Table 2. In Doppler records, QT is equivalent to Q-Ac.

**Table 1:** Comparison between the estimated and reference values of QT and QTc intervals for the normal and abnormal nfECG records.

Category	Number of beats	RR (ms)	HR (bpm)	QT (ms)			QTc (ms)		
				Reference	Model-based	RMSE	Reference	Model-based	RMSE
*Normal*	17,227	425 ± 28	142 ± 9	245 ± 15	244 ± 16	10	326 ± 15	325 ± 14	13
*Abnormal*	1,883	415 ± 59	147 ± 20	253 ± 30	242 ± 34	17	339 ± 26	324 ± 30	23

**Table 2:** Detailed summary of the abnormal case results.

Category	Number of subjects(number of beats)	RR (ms)	HR (bpm)	QT (ms)			QTc (ms)		
				Reference	Model-based	RMSE	Reference	Model-based	RMSE
*Bradycardia and LQTS*	1 (200)	557 ± 8	108 ± 2	325 ± 9	324 ± 7	9	395 ± 11	394 ± 7	11
*Tachycardia*	2 (294)	334 ± 22	181 ± 13	218 ± 10	196 ± 9	22	314 ± 10	283 ± 6	32
*HeartAnomaly*	2 (645)	405 ± 8	148 ± 3	256 ± 10	237 ± 6	21	347 ± 14	321 ± 7	29
*IUGR*	1 (333)	432 ± 8	139 ± 2	248 ± 11	254 ± 10	10	328 ± 15	336 ± 12	13
*VasaPrevia*	1 (225)	406 ± 10	148 ± 4	239 ± 9	233 ± 6	12	323 ± 12	315 ± 6	16
*HeartFailure*	1 (13)	408 ± 4	147 ± 1.3	243 ± 13	231 ± 2	16	327 ± 17	311 ± 2	22
*Placental Dysfunction*	1 (173)	411 ± 8	146 ± 3	247 ± 9	237 ± 8	12	333 ± 12	318 ± 9	16

**Fig 5:**
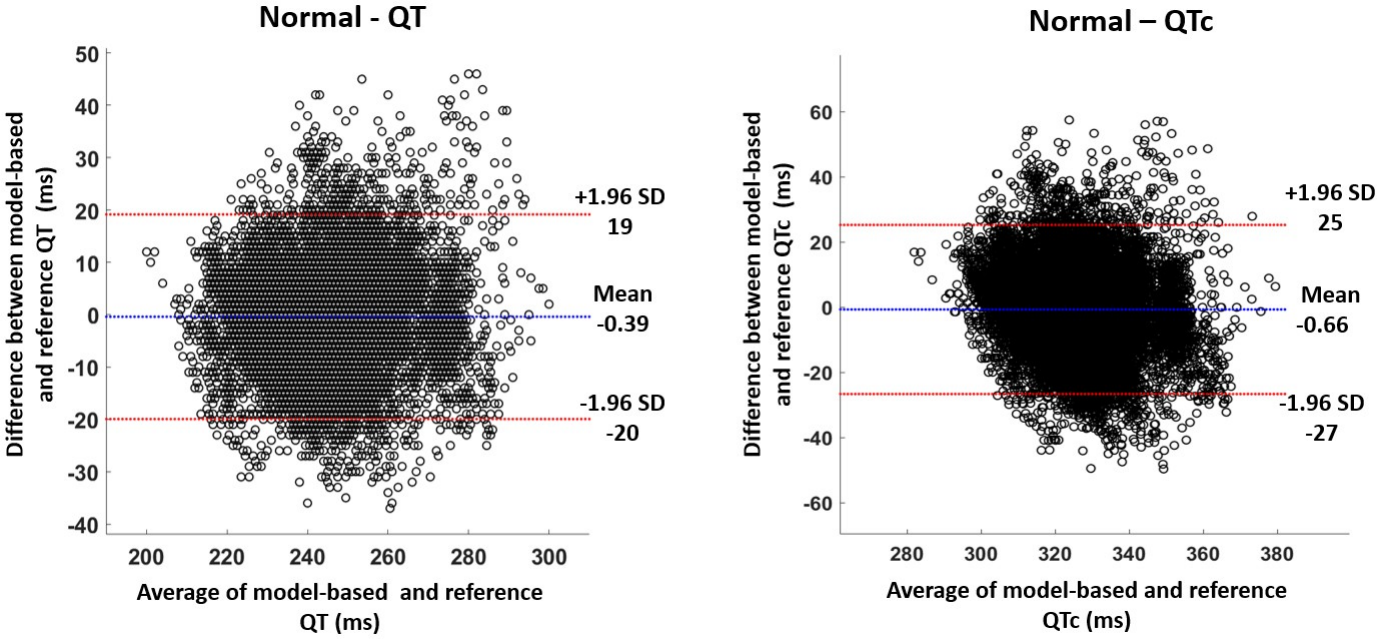
The figures show Bland Altman plots for QT (left) and QTc (right) intervals of normal subjects. The total number of points in both figures is 17,227. The total number of points that fall within the LoA is 16,367 (95%) and 16,378 (95%) for the QT and QTc intervals respectively. LoA: Limits of agreement.

**Fig 6:**
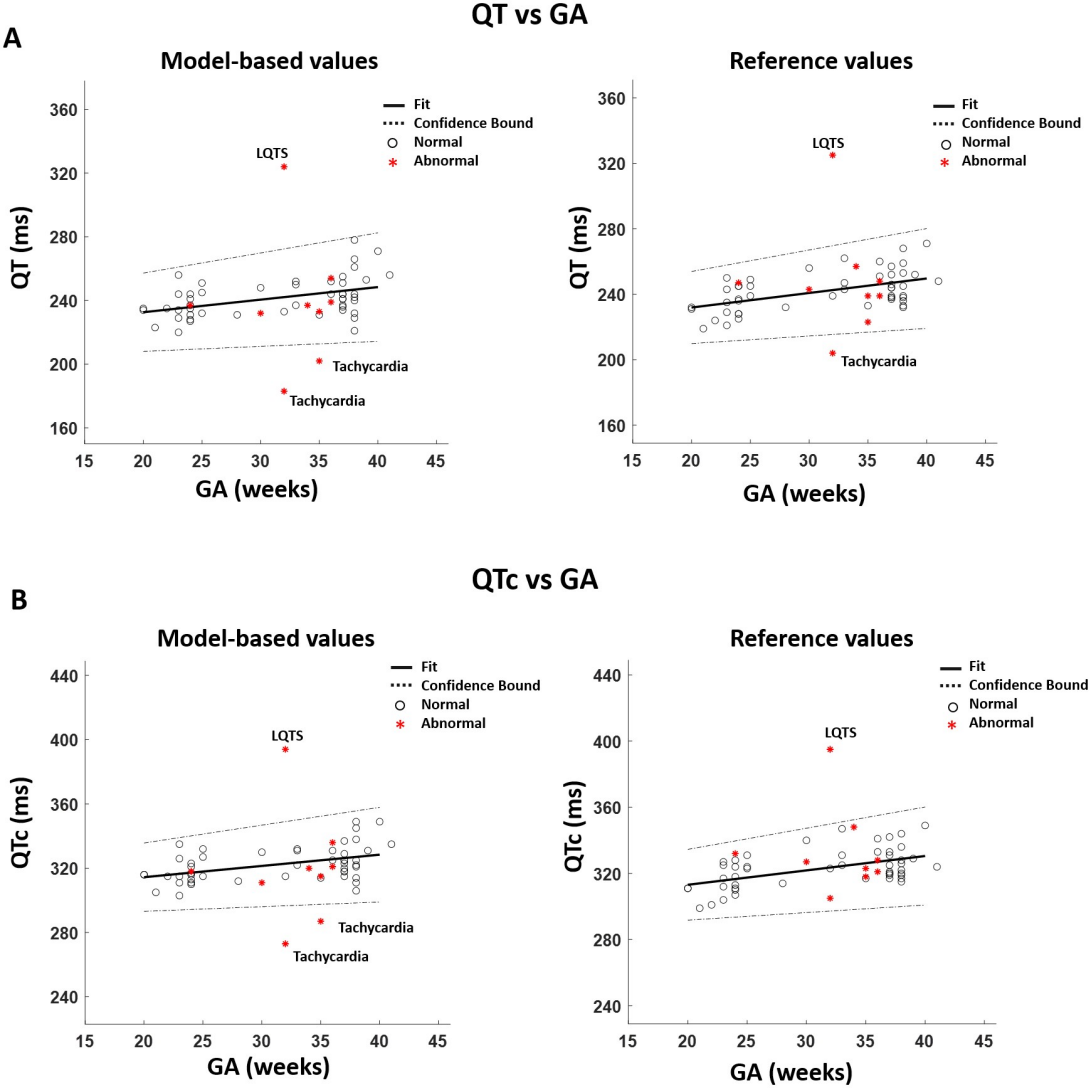
The figures show estimated and reference QT and QTc values plotted against GA. The normal cases are shown in circle and the abnormal cases are shown in asterisk. Both model-based and reference values show linear increasing trends. In (A), LQTS and tachycardia are outside the confidence bound in the model-based values plot. On the other hand, LQTS and one case of tachycardia are outside of the confidence bound in the reference values plot. In (B), LQTS and tachycardia are outside the confidence bound in the model-based values plot. In the reference values plot, only the LQTS case is outside the confidence bound. LQTS = Long QT Syndrome.

Bland Altman plots [40, 41] were calculated to measure the degree of agreement between the estimated and reference values of QT and QTc intervals. The Bland Altman plots of QT and QTc intervals for the normal cases are shown in Fig 5. In Fig 5, the total number of points that fall within the limits of agreement (LoA) is 16,367 (95%) and 16,378 (95%) for QT and QTc intervals, respectively. The mean of QT and QTc for the normal values have been plotted against GA as shown in Fig 6. The abnormal values have been included in the plot to compare them with the normal values. Fig 6 shows an increasing trend between both of QT and GA and QTc and GA.

## Discussion

**Fig 7:**
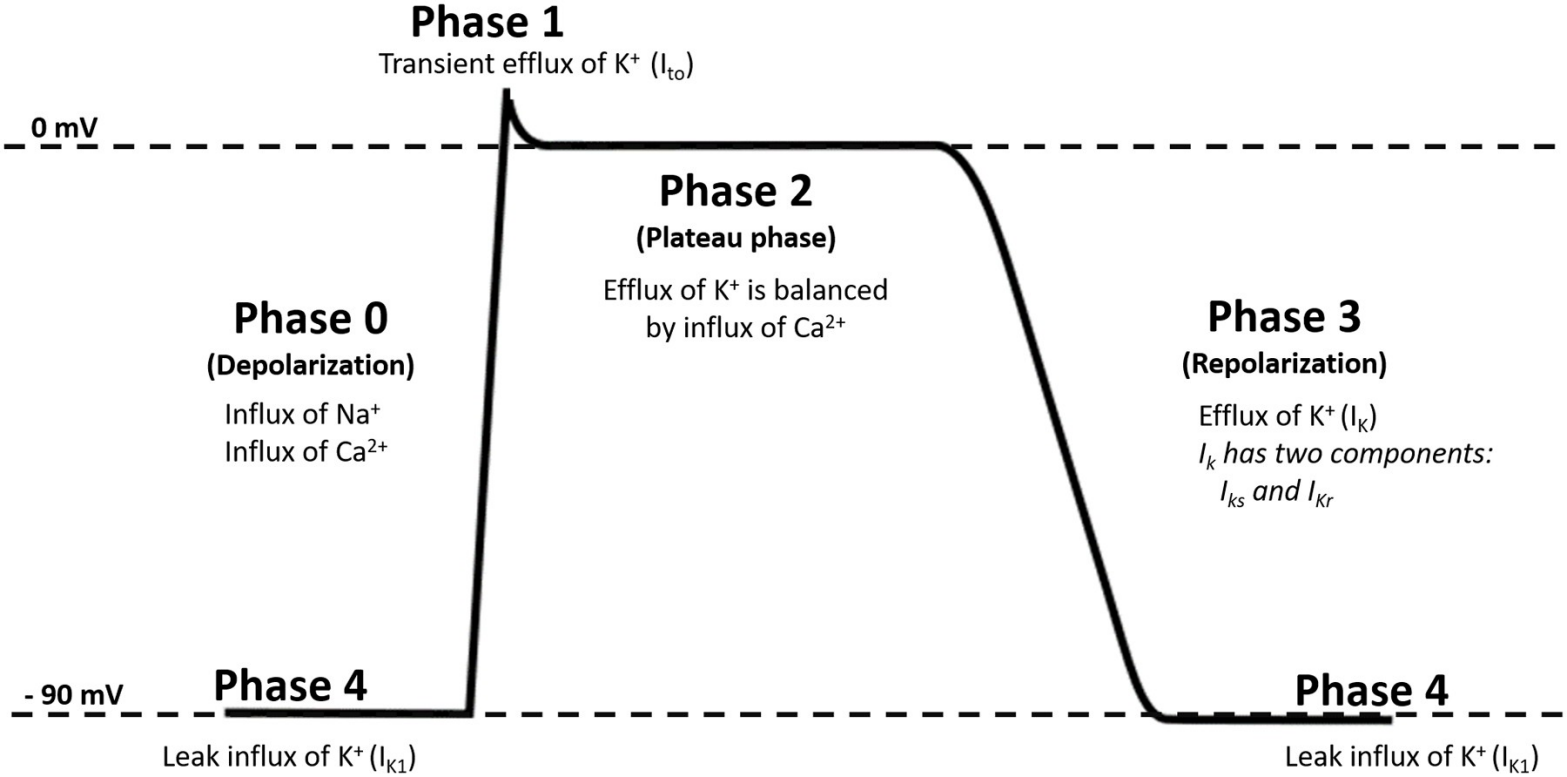
Ventricular AP. During depolarization or phase 0, Na^+^ and Ca^+^ channels open causing influx of Na^+^ and Ca^+^. The depolarization phase increases cellular potential. When the cellular potential reaches a certain level, specific K^+^ channels open causing a transient flow of K^+^ out of the cell, phase 1. The transient current is known as I_to_. The efflux of I_to_ causes a slight reduction in the cellular potential. Phase 1 is followed by phase 2 or plateau phase in which the efflux of I_to_ is balanced by the influx of Ca^2+^. In phase 3, more K^+^ channels open to restore the cell into the resting potential. The current in phase 3 is known as I_k_ and it has two components, rapid (I_kr_) and slow (I_ks_). The efflux of I_k_ current continues until the cell is restored to its resting potential in which the dominant current is a leak K^+^ current known as I_k1_.

The ventricular repolarization phase in AP is mainly dominated by the action of K^+^ channels [18, 42–44]. Throughout the AP, different types of K^+^ channels are activated to regulate the flow of K^+^. After a depolarization occurs, a transient current of K^+^ (I_to_) starts flowing outward of the cell [18]. As AP progresses, the magnitude of the K^+^ flowing outwardly increases due to the opening of more K^+^ channels. The latter current is denoted as the delayed rectified potassium current (I_K_) [18]. I_K_ flows during Phase 3 and is composed of a slow current (I_ks_) and a rapid current (I_kr_). During phase 4, another type of K^+^ current flows inwardly (I_k1_) causing hyperpolarization [18]. The ventricular AP is summarized in Fig 7.

*H. Konarzewska et al*. [42] conducted a study to see the difference between the left ventricular subepicardial myocytes and the right septal subendocardial myocytes in terms of the magnitudes of I_k_, I_k1_ and I_to_ currents. The study was conducted on biopsy samples collected from human subjects. *H. Konarzewska et al*. [42] studied the different magnitude of currents by finding the relationship between the currents and different applied voltages. The time course obtained for I_to_ and I_k1_ inactivation were fitted with exponential equations. The equation obtained for I_to_ inactivation was I_to1_(t) = A_0_ + A_1_ e^−t/T^. The latter equation is similar to the equation obtained in this study. Another study performed by *K. Furutani et al*. [43] discusses a model for I_K_. *K. Furutani et al*. [43] mention two equations that model the activation and inactivation of the I_K_ channels and both equations have exponential expressions on them.

The model in this study has been developed based on our knowledge of AP of human adults. To our knowledge, there is no previous research that addresses AP in human fetuses, therefore, it is unknown how AP looks like in human fetuses and how it changes with GA. Several previous research papers addressed changes of ventricular AP with GA in animal subjects. *S. Hamaguchi el al*. [45] and *J. Couch el al*. [46] show that rats and mice fetuses exhibit a slightly different AP than adult AP. Phases of AP (0-4) in prenatal rats/mice were visible in early GA of fetal mice/rats. As the fetuses grew, phase 2 (plateau phase) of AP decreased until it disappeared. In neonatal and adult mice/rats, there is no phase 2 [46]. The fact that the plateau period decreases at late GA may explain the reason behind increased HR in adult mice/rats [47]. The repolarization phase in mice/rats showed exponential decays during various GA.

*T. Huynh et al*. [48], discuss how AP changes in fetal, neonatal and adult rabbits. In contrast to mice/rats, the study demonstrates that the plateau phase increases with GA. In addition, similarly to mice/rats, the repolarization phase in rabbits shows exponential decays. Based on the research done on animal subjects, one can assume that AP of human fetuses may exhibit similar trends to rabbits and opposite to mice/rats. Therefore, one can assume that the AP of human fetuses increases with GA and the repolarization phase exhibits an exponential decay. In fact, the results in Fig 6 further supports the assumption that the AP increases with GA. The fact that QT and QTc increase with GA is also addressed in [49, 50].

The model developed in this study has been used to identify end of T waves in normal and abnormal subjects. The results in Table 1 show that the mean values for the estimated and reference values are close in the normal and a little bit different in the abnormal case. The results of the model in normal subjects have been validated by Bland Altman analysis, Fig 5. The Bland Altman test shows that at least 95% of the data falls within the confidence bound for both QT and QTc. For further validation, the mean values of QT and QTc for each subject has been plotted against GA in Fig 6. The plot shows that all the normal subjects have differences of less than 5% between the model-based and reference values.

Since there are no reference values for normal fetal QT and QTc [11], the values obtained in this study have been compared with previous literature. *C. Velayo el al*. [2] report fHR, QT and QTc values of (149 ± 9) bpm, (234 ± 23) ms and (370 ± 40) ms, respectively, for 20 healthy fetuses (GA: 20-33 weeks). *C. Velayo el al*. [2] measured QT by identifying the Ac timings in Doppler signals. Another study by *A. Khandoker et al*. (23), reports RR interval and Q-Ac values of (421 ± 33) ms and (225 ± 13) ms, respectively, for 21 healthy fetuses (GA: 28-36 weeks). *N. Sato et al*. [50] report values for normal fECG during the active and resting phases (GA: 18-41 weeks). The reported values of fHR, QT and QTc during the active phase are (150 ± 1.7) bpm, (244 ± 3.2) ms and (384 ± 4) ms, respectively (n = 31, GA = 34 ± 2.9 weeks). During the resting phase, the reported values are (139 ± 1.1) bpm, (246 ± 2.8) ms, and (374 ± 4) ms, respectively (n = 29, GA = 35 ± 0.6 weeks).

*J. Stinstra et al*. [49] measured several fECG features from a total of 582 healthy fetuses (GA: 29—34 weeks) using fMCG within several medical centers. Different values of QT and QTc have been reported for each center. The total number of subjects that had their QT and QTc evaluated was 412 and 274, respectively. The ranges of values that are reported for QT and QTc are ((227 ± 12) ms—(255 ± 13) ms) and ((370 ± 10) ms—(400 ± 20 ms)), respectively. Another study done by *S. Abboud et al*. [51], reports values of QT with a min of 205 ms, a max of 338 ms and an average of (255 ± 28) ms for 17 fetuses (GA: 32-41 weeks). The values of normal QT obtained for this study, Fig 6, fall within the ranges mentioned in the previous literature. The above-mentioned literature has used Bazett’s formula [29] for QTc calculations. Therefore, the obtained values in this study for QTc cannot be compared with them because a different formula has been used.

The model has been developed mainly to predict end of T waves in normal subjects. The abnormal subjects have been included in order to test how the accuracy of the model changes with the presence of abnormalities. The results in Table 2 show variations in accuracy among the abnormal cases. The highest accuracy is observed for the LQTS case (QT RMSE = 9 ms and QTc RMSE = 11 ms). The least accuracies are observed for the tachycardia (QT RMSE = 22 ms and QTc RMSE = 32 ms) and heart anomaly (QT RMSE = 21 ms and QTc RMSE = 29 ms) cases. The high accuracy for the LQTS implies that this model could be a good estimate for QT and QTc in fetuses suffering from LQTS. The plots in Fig 6A show that tachycardia and LQTS fall outside the confidence interval in the model-based plot. On the other hand, LQTS along with one case of tachycardia are outside the confidence bound in the reference values plot. Fig 6B shows that LQTS and tachycardia are outside the confidence bound in the model-based values plot. In the reference values plot, only LQTS is outside the confidence bound. The separation of LQTS from the normal subjects was evident in both the model-based and reference values plots. LQTS occurs mainly due to mutations in the genes responsible of encoding the pore-forming *α*-subunits of some of the ion channels responsible of the AP [52]. The affected channels could be the channels responsible of the regulation of I_kr_, I_ks_ or ${\text{I}}_{{\text{Na}}^{+}}$. The latter channels are also expressed in the sinoatrial node (SAN). Therefore, LQTS are usually present in individuals with abnormal HRs [52]. The LQTS case in this study suffers from bradycardia as well. Since the model estimates the end of T wave based on RR, it could calculate the end of T waves for the LQTS case.

The tachycardia results in Fig 6 were different in the reference and model-based values which further confirms the inability of the model of evaluating T wave ends in tachycardia cases. The values provided by the model indicates that the QT and QTc intervals of tachycardia should be shorter than normal, since they were less than the lower confidence bound. On the other hand, the reference values plots show, mostly, that the QT and QTc intervals for fetuses with tachycardia are normal since they are within the confidence bound. Although the model was good for LQTS and bad for tachycardia, it is hard to draw conclusions about the validity of the model for the LQTS case. The number of subjects in both cases was low, therefore, the model should be applied on more subjects, specially, subjects who have high HRs with LQTS.

The model developed in this study shows good results for the normal cases. Nevertheless, there are some limitations with the study. Most of the beats in this study have been validated using Doppler signals. Therefore, more accurate methods should be used for the validation of the results. Since fsECG signals are limited to late GA, M-mode Doppler images could be a better method of validation. Another limitation is the inability to measure the location of Q peaks from Doppler records directly. It would have been more accurate to compare Q-Ac from Doppler signals with QT from nfECG records.

## Conclusion

End of T waves are important for QT estimation which are biomarkers for many cardiac complications including sudden cardiac death. However, identifying the end of T waves in nfECG is challenging due to the high level of noise as compared to T wave amplitude. In this study, a novel method for estimating the end timings of T waves based on RR intervals in nfECG has been discussed. The model showed high agreement with reference values in healthy as well as some unhealthy fetuses. The highest accuracy was observed in a fetus suffering from bradycardia and LQTS showing the prolongation of QT intervals. On the other hand, the least accuracies were observed for tachycardia and heart anomaly cases. The good results obtained for the normal fetuses imply that the model is effective in predicting T wave end timings in nfECG records of normal fetuses. Therefore, the model can be used for the prediction of a QT interval non-invasively from RR interval. However, this technique needs further validation on a large number of normal and LQTS cases in future clinical studies.
